# The Dynamic Relationship between the Glymphatic System, Aging, Memory, and Sleep

**DOI:** 10.3390/biomedicines11082092

**Published:** 2023-07-25

**Authors:** Konstantinos I. Voumvourakis, Eleni Sideri, Georgios N. Papadimitropoulos, Ioanna Tsantzali, Paul Hewlett, Dimitrios Kitsos, Marianna Stefanou, Anastasios Bonakis, Sotirios Giannopoulos, Georgios Tsivgoulis, George P. Paraskevas

**Affiliations:** 1 2nd Department of Neurology, School of Medicine, “Attikon” General University Hospital, National and Kapodistrian University of Athens, 12462 Athens, Greece; cvoumvou@outlook.com (K.I.V.); elenisideri1985@gmail.com (E.S.); bonakistasos@med.uoa.gr (A.B.);; 2 Applied Psychology Department, Llandaff Campus, Cardiff Metropolitan University, Western Avenue, Cardiff CF5 2YB, UK

**Keywords:** memory, aging, glymphatic system, molecular system, neuroinflammation

## Abstract

The process of memory entails the activation of numerous neural networks and biochemical pathways throughout the brain. The phenomenon of memory decline in relation to aging has been the subject of extensive research for several decades. The correlation between the process of aging and memory is intricate and has various aspects to consider. Throughout the aging process, there are various alterations that take place within the brain and, as expected, affect other functions that have already been linked to memory and its function such as involving microcirculation and sleep. Recent studies provide an understanding of how these mechanisms may be interconnected through the relatively new concept of the glymphatic system. The glymphatic system is strongly correlated to sleep processes. Sleep helps the glymphatic system remove brain waste solutes. Astrocytes expand and contract to form channels for cerebrospinal fluid (CSF) to wash through the brain and eliminate waste. However, the details have not been totally elusive, but the discovery of what we call the glymphatic system enables us to connect many pieces of physiology to understand how such factors are interconnected and the interplay between them. Thus, the purpose of this review is to discuss how the glymphatic system, sleep, memory, and aging are interconnected through a network of complex mechanisms and dynamic interactions.

## 1. Introduction

Comprehending the underlying mechanisms and potential interventions related to cognition and brain health requires a thorough understanding of the interaction and interconnection between memory, aging, sleep, and the glymphatic system. Memory, which involves the stabilization and storage of memories, is subject to influences such as age-related alterations in the brain [1]. The process of aging is accompanied by a range of neurobiological alterations in the brain, which entail an accumulation of toxic proteins, such as beta-amyloid plaques and tau tangles, as well as a decrease in brain volume and synaptic connections. These changes have the potential to affect the functionality of cerebral areas associated with memory, such as the hippocampus and prefrontal cortex, which lead to memory loss. In addition, the process of aging is accompanied by a decline in energy production, as well as an increase in oxidative stress and mitochondrial dysfunction .This may cause injury to cellular functions and memory loss [2]. Besides this effect, another interaction of aging is the changes in sleep. Aging causes frequent modifications in sleep patterns, such as a reduction in sleep efficiency, heightened wakefulness throughout the night, and modifications in sleep architecture [3]. Changes in sleep patterns may have an impact on the process of memory consolidation and overall cognitive functioning. There is a wealth of evidence suggesting that sleep plays a significant role in this phenomenon, primarily due to the distinct neurochemical milieu and electrophysiological patterns observed during the sleeping period. Two hypothetical models, which may not be mutually exclusive, have been suggested to account for the valuable impact of sleep on memory functions. These models are referred to as the hippocampal–neocortical dialogue and the synaptic homeostasis hypothesis [4]. Both sleep patterns and aging can affect the glymphatic system’s functionality [5]. During sleep, specifically slow-wave sleep (SWS), the glymphatic system is at its most active. During SWS, the interstitial space within the brain enlarges, allowing the glymphatic system to flush out accumulated waste products [6,7]. Typically, older adults experience diminished SWS, diminished sleep efficacy, and increased sleep fragmentation [8]. The activation and process of the glymphatic system may be impacted by these age-related changes in sleep patterns. Also associated with aging are modifications to the glymphatic system itself [9]. Studies suggest that the effectiveness of glymphatic clearance declines with age, which may contribute to the accumulation of waste products in the brain [9,10]. Changes in the structural integrity of glymphatic vessel networks and the modulation of specific molecules [11], alongside age-related vascular changes such as diminished cerebral blood flow and an increased permeability of the blood–brain barrier [12], are potential factors that may contribute to the compromised glymphatic function observed in older individuals. Consequently, sleep disturbances and impaired glymphatic function can impede the efficient clearance of metabolic waste products, such as beta-amyloid [13]. This accumulation of waste products can negatively impact brain health, lead to memory loss, and increase the risk of neurodegenerative diseases such as Alzheimer’s disease (AD) [14]. Combined with age-related changes in sleep patterns, the impaired glymphatic function observed with aging may contribute to the increased prevalence of neurodegenerative diseases in older individuals [13,14].

## 2. Memory, Aging, and Sleep Interaction

Memory is essential for cognitive ability, personal identity, and emotional well-being. It enables us to learn, adapt, and navigate the environment around us. Memory can at the same time be thought of as a behavior, a process, a brain function, or even a model of neural activity [15]. Memory is multimodal and is comprised of long-term memory (LTM), short-term memory, and working memory [16]. Long-term memory is formed and maintained by neuronal ensembles composed of excitatory and inhibitory neurons, which undergo synaptic and neuronal modifications at various stages of their formation [17,18]. Long-term potentiation is a crucial process that responds to memory-related stimuli and induces brain-activity-induced changes. Other cell types, such as astrocytes and microglia, have been found to play a role in memory regulation [19]. Long-term memory is formed and stored in the hippocampus, notably the dentate gyrus and Cornu Ammonis (CA) regions 1 and 3. Schaffer’s collaterals are critical to long-term potentiation memory storage [20]. AMPA, NMDA, and glutamate receptors enhance action potentials and calcium and sodium ion exchange to facilitate this process [21]. Although the investigation of memory is very demanding and complicated, researchers have made progress in elucidating the structures, processes, and molecular systems involved in the formation and consolidation of long-term memory. However, there is still a need for the identification of the effects of factors such as sleep and aging [22]. Long-term memory and short-term memory are interconnected through the processes of the formation and retrieval of memories. Together, they facilitate the storage and retrieval of information in the human brain [23]. Short-term memory relies on alterations in synaptic strength among pre-existing neuronal connections through the covalent modification of proteins, particularly enzymes of the kinase family [24,25]. Key enzymes involved in this process include A-protein kinase (APK), protein kinase C (PKC) [25], calcium/calmodulin-dependent protein kinase II (CaMK II) [26], and mitogen-activated protein kinase (MAPK) [27]. These proteins play important roles in modulating synaptic plasticity and the consolidation of short-term memory [25]. Working memory uses frontoparietal brain regions like the parietal cortices, prefrontal cortex, and cingulate gyrus. Working memory and protein dephosphorylation depend on the prefrontal cortex and other cortical areas [28]. Intracellular signaling pathways, particularly involving calcium- and cAMP-dependent protein kinases, can impair working memory [29]. Understanding working memory neural mechanisms and variables is crucial to comprehend cognitive processes and manage memory deficits or declines in diverse circumstances (please see Table 1).

Aging has significant effects on memory, starting at the molecular level. These effects of aging are interconnected and thus influence one another [30]. Cellular senescence, which increases with age as the name implies, affects neuronal function and contributes to chronic inflammation [31]. Mitochondrial dysfunction impacts the energy required for cellular processes, leading to neuronal injury and memory loss associated with aging. Aging also induces synaptic changes, including a decline in synaptic density and a shift in excitatory–inhibitory neurotransmission [32]. Inflammation interferes with synapse function as well as memory development and retrieval [33]. Protein aggregation is linked to aging and cognitive impairment, and it is hypothesized to be important in the etiology of several neurodegenerative diseases [34,35]. Memory loss and the dysfunctions inherent in aging are critical and correlate with the way the brain processes biomolecules or removes likely toxic metabolites, produced through neuronal activity and interaction [36]. The clearance of these molecules is obviously vital, particularly in the case of the amyloid beta (Aβ) peptide, a byproduct mostly of membrane maintenance, which negatively affects neurons by coalescing into amyloid plaques, a key factor in the pathogenesis of AD and neurodegeneration in general [37,38]. The interplay between synaptic activity, mitochondrial function, and sleep is crucial in maintaining a healthy brain [39]. Sleep abnormalities can disrupt the typical energy metabolism of cells, including those located within the brain. Sufficient rest is essential for mitochondria to effectively synthesize adenosine triphosphate (ATP), which serves as the primary energy unit for cellular processes [40,41]. Sleep deprivation may affect the capacity of mitochondria to produce ATP, resulting in decreased cellular energy availability [42].

Sleep is one of the most important elements in human homeostasis. The interlinkage of sleep and brain functionality is provided through circuits that are interacting parts of a wider system generated by sleep’s multiple properties and interactions, including changes in memory, rhythmic brain dynamics, regulated respiratory and circulatory physiology, and clearance [43]. Studies in memory and sleep correlation have recorded individual brain areas and cell types, showing sleep-regulating areas in the hypothalamus, brainstem, basal forebrain, and other subcortical nuclei [44]. As mentioned above, sleep deprivation can lead to compromised mitochondrial function, resulting in a decrease in ATP production, which may have a detrimental impact on memory-related processes. An inadequate amount of sleep can impede the appropriate execution of mitophagy, resulting in the buildup of impaired mitochondria. The accumulation of such substances has the potential to compromise the overall functionality of mitochondria and intensify any pre-existing mitochondrial impairments [45]. Dysfunctional mitophagy has been implicated in the pathogenesis of several age-related diseases due to sleep deprivation or abnormalities, including neurodegenerative disorders such as Parkinson’s disease and Alzheimer’s disease [45,46]. The discovery that sleep helps remove waste from the brain at far higher rates than during wakefulness is vastly significant due to it furthering our understanding of interstitial fluid dynamics. Sleep thus becomes a part of the removal mechanism of potentially damaging metabolic waste from neurons [47]. As individuals age, they often experience changes in their sleep patterns, with elderly people experiencing more awakenings at night and less efficient sleep. It is also possible to develop the advanced sleep phase syndrome due to changes in bedtime hours. As a consequence, sleep architecture variations due to aging affect memory and brain function.

Therefore, the relationship between aging and sleep is bidirectional. Age-related changes in brain structures, such as the suprachiasmatic nucleus (SCN) responsible for regulating the sleep–wake cycle, can disrupt the circadian rhythm. Additionally, molecular and cellular changes associated with aging, such as inflammation and hormonal alterations, can influence sleep patterns [48]. Poor sleep correlates with a cortical Aβ burden and CSF A and phosphorylated tau levels in elderly people with AD [49]. Patients with mild cognitive impairment (MCI) and AD have significantly less posterior NREM sleep than healthy older adults, with the degree of reduction predicting the severity of memory impairment [50]. Adverse post-mortem investigations have shown that neurofibrillary tangles in the preoptic region of the hypothalamus correlate with the severity of prior decreased sleep. Tau deposition is also observed in cognitively normal older adults in the locus coeruleus and basal forebrain, leading to the currently untested hypothesis that tau within these regions may induce sleep abnormalities years before the onset of degenerative disease and serve as an early diagnostic biomarker [49,50].

## 3. Neurofluids and Memory

### 3.1. The Glymphatic System and Its Wider Scope

The proper regulation of the CNS milieu is vital for its healthy functionality. Imbalance in the concentrations of critical ions and other soluble molecules, neurotrophic factors, and waste is both the result and the cause of potentially severe dysfunction. The removal of toxic substances, such as metabolic waste, from the brain is necessary to maintain its proper functioning throughout life. 

The idea of a net flow of extracellular fluid driving the interstitial clearance of waste and various other solutes in the brain is by no means new. Clearance by diffusion alone, after all, would lead to concentration gradients of solutes across the parenchyma. This would be at odds both with observed clearance rates and the assumed homeostasis in the microenvironment surrounding the brain cell [51]. However, it was not until 2012 that comprehensive evidence for a global, effective mechanism was presented. Termed by Iliff et al. as a “glymphatic” system, a portmanteau of glial and lymphatic [52], this mechanism was proposed to affect a bulk convective flow of fluid through the extracellular space (ECS) of brain parenchyma. Through the utilisation of in vivo two-photon excited fluorescence microscopy observations, a carefully planned study design, and a strong execution, the researchers successfully illustrated the transportation of tracer molecules with different molecular weights across the different components of the system. This process initiated with the cerebrospinal fluid (CSF) penetrating deeply into the parenchyma, and then being carried along the perivascular spaces known as Virchow-Robin spaces (VRS).The interstitial flow was theorized to be driven by the influx of periarterial CSF towards the ECS through the aquaporin-4 channels that are heavily concentrated (“polarized”) at the astrocytic end feet of the glia limitans, which line the perivascular spaces of the penetrating arterioles. This influx ultimately amounts to a bulk current of advection towards the perivenular spaces of deep venules, from where the soluble waste is drained away towards the cervical lymphatic and venous circulation.

In this manner, the advection flow serves as a compensatory mechanism for the significantly limited lymphatic drainage within the parenchyma. Moreover, it addresses the sluggish dynamics of extracellular fluid in the protected cerebral interstitium. A significant characteristic of the protected cerebral interstitium is that it exhibits lower turnover rates compared to organs lacking a blood–parenchyma barrier. In doing so, this mechanism integrates much that is already known or suspected in our investigations on brain waste clearance, the role, and functions of CSF/ISF, as well as the perivascular spaces in health and disease [53,54,55]. 

However, despite its elegance as a concept and the quality of the supporting experimental data, the theory of glymphatic flow was met with some skepticism over key aspects of its proposed function. It is exceedingly difficult to directly image in vivo its components or processes involved. Such processes, also, are very hard to quantify in vivo, ex vivo, or even in silico, where for example, computational models for advection through the complex geometry of the cerebral parenchyma refute the idea of macromolecule lavage [56]. Controversy exists even on a much more accessible level, regarding the fluid mechanics of the perivascular space and the role of arterial pulsations in its function as an effective deep brain conduit for CSF [57]. 

Over more than ten years since its inception, however, the theory for a glymphatic system has been proven to be a major breakthrough and has stimulated a renewed and vigorous interest in the homeostasis of brain fluids. Several advances in our understanding of parenchymal physiology, the role of Aquaporin 4, the interstitial fluid dynamics during the wake–sleep cycle, and the discovery of cerebral lymphatics along the dural venous network have provided the groundwork for a more solid integration of the glymphatic system hypothesis in the wider scope of intracranial circulation. The term neurofluids has been coined to encompass these interconnected yet compartmentalized fluid reservoirs that lie at the center of the circulatory physiology of the CNS [58,59], thus endorsing the implication of the dynamic interrelation of physical and biomolecular mechanisms in what has been an archipelago of fragmentary knowledge.

### 3.2. Glymphatic System and Brain Functionality

The proper regulation of the CNS milieu is vital for its healthy functionality. Imbalance in the concentrations of critical ions and other soluble molecules, neurotrophic factors, and waste is both the result and the cause of potentially severe dysfunction. The removal of toxic substances, such as metabolic waste, from the brain is necessary to maintain its proper functioning throughout life. Recently, two correlated and dynamic networks have been discovered in the brain for the removal or clearance of toxic substances and brain fluids as a critical component in brain tissue homeostasis [7]. The brain and spinal cord show disproportionately high metabolic rates and synaptic transmission is extremely sensitive to homeostatic changes [60]. Thus, the central nervous system, which has no lymph vessels, has developed unique adaptations to achieve homeostasis through the turnover of fluids and the removal of toxic intermediates through the glymphatic system [61]. Change it to “The glymphatic system comprises and uses a perivascular network for the transport of CSF” [52,62]. This system is theoretically connected with the functions of the lymphatic network and has been connected to the meninges (dura), cranial nerves, and the large vessels of the cranium [63,64]. The anatomical and functional components of these systems are complex and the procedures by which they are interconnected are not yet clearly understood. Studies focused on glymphatic system processes suggested that it contributes to the transportation of amyloid-beta and tau oligomers out of the brain through the turnover of the interstitial fluid (ISF) [47]. CSF flows through the periarterial spaces, basal cisterns, and subarachnoid space and envelops the cerebral hemispheres; CSF is driven from the periarterial system into the area of ISF. In this phase, ISF is aided by aquaporin 4 (AQP4) water channels on astroglia, combining CSF-ISF and enhancing the waste solute removal [65]. The next step after the intermixing of interstitial waste solutes into CSF-ISF is their transportation towards the perivenous region of the larger central veins, where it drains to the LVs, thus completing the circuit of the parenchymal perivenous outflow of the brain towards the lymphatic circulation [10,52]. Aging has an impact on the AQP4 water channel procedures. Specifically, the AQP4 polarization on the astroglia processes encircling the cortical-penetrating arterioles is shown to be decreased in older subjects’ brains compared to younger subjects, as was firstly demonstrated in animal model protocols [10]. Aging and a plethora of chronic diseases affect lymphatic drainage [65,66]. In neurodegenerative diseases such as AD and to a lesser extent in physiological aging, the glymphatic system is affected as well. The dysregulation of AQP4 expression in astrocyte end feet is a proposed mechanism for this [66,67], independently or within other pathological processes. An example of this is reactive astrogliosis secondary to an inflammatory offense, giving rise to a positive feedback loop of the impaired clearance of metabolites [68,69] and accumulation of cytokine and other inflammation mediators [69,70,71], which in turn exacerbates neuroinflammation, providing the putative link between microglial and astrocyte reactivity and neurodegeneration.

### 3.3. Glymphatic System, Aging, and Memory

The clearance mechanisms of compounds that are implicated in neurodegenerative diseases have naturally been major foci of research, which has already lent us priceless insights regarding the roles of the various compartments of neurofluid circulation [67]. Indeed, Aβ amyloid accumulation, which directly leads to memory impairment, seems to correlate to the brain’s glymphatic–lymphatic system’s inability to affect normal waste drainage. As a consequence, the effects of aging on the meningeal lymphatic vessels that affect the glymphatic–lymphatic clearance of toxic substances, including Aβ amyloid, constitute a major risk factor for developing neurodegenerative diseases such as AD [55,67,72]. An investigation of the glymphatic system reveals that an enhancement of this system can decrease the solute Aβ in the brain parenchyma and delay brain aging; however, this did not ultimately affect the formation of amyloid plaques. Relevant studies in AD models have suggested that reducing the deposition of beta-amyloid (Aβ) [73] and tau protein can improve memory, and increase the internalization of Aβ in microglia, indicating that the unimpeded function of the glymphatic system may significantly contribute to the conservation of memory functions as aging progresses [74]. Another suggested repercussion of impaired glymphatic system function is the amplification of neuroinflammation through reduced cytokine clearance in the brain as shown above. We have already seen that cytokine receptors are found in neurons, microglia, and astrocytes and affect structural differentiation at the synaptic level. Hence, inflammation, through the effects of cytokine activity, aggravates memory decline in a more direct and specific way and affects the plasticity of the brain [52,75]. Glymphatic system impairment is involved in vascular dementia as well. Cognitive dysfunction has been associated with decreased neuronal myelin in the context of white matter damage and infarcts, as observed in vascular dementia, frequently involving the perivascular spaces, damaging water channels, and Aquaporin-4 activity around blood vessels. This presents an additional mechanism of glymphatic insufficiency through the impairment of CSF reabsorption across perivascular pathways, consequently impairing waste clearance from the brain [52]. 

### 3.4. Sleep and Glymphatic Interactions

The underlying biological need for sleep is unknown, despite multiple studies showing that sleep improves memory consolidation, which may be vital for competition among species [76]. The fact that the brain’s energy metabolism only drops by 25% while sleeping suggests that the primary function of sleep is not energy conservation [77]. New research indicates that glymphatic activity is greatly increased during sleep but repressed during waking. Advanced imaging methods revealed a 90% decrease in the CSF influx in awake mice compared to those who had been sedated [51]. The same experiment was carried out on normally sleeping mice to determine if the results were unique to the unconscious condition or a result of the anesthetics [77,78]. Other investigations of CSF influx demonstrated a striking resemblance between real sleep and anesthetized animals. This observation shows that the sleep state is particularly favorable to convective fluid fluxes and consequently to the clearance of metabolites [77]. The activation of the glymphatic system and the removal of neurotoxic waste products from the brain during sleep thus appear to be two of the most important functions of sleep [6]. The fact that glymphatic function is very active in both anesthetized and naturally sleeping mice but not in awake animals suggests that differences in sleep vs. wakefulness, rather than daily circadian rhythms, drive glymphatic activity [79]. The neuromodulator norepinephrine is a primary arousal inducer. Norepinephrine also reduces CSF synthesis by acting directly on choroid plexus epithelial cells. In contrast, blocking norepinephrine signaling, which mimics the sleep state, increases CSF production [77,80]. The coordinated action of norepinephrine thus acts via various mechanisms on both fluid availability and convective fluxes to decrease glymphatic function, and norepinephrine can thus be considered a fundamental regulator of both the sleep–wake cycle and solute clearance from the brain [75,80]. Glymphatic clearance occurs mostly during slow-wave sleep, also known as the N3 sleep stage. Slow-wave sleep has been associated with both glymphatic clearance and dementia [6]. A third of AD patients have clinically documented sleep problems, and the great majority of them have a reduced total sleep time and decreased slow-wave sleep, both of which commonly precede the development of the disease [81]. Alzheimer’s disease affects the intricate cascade of neurotransmitters and hormones involved in sleep regulation [73,79]. Furthermore, a single night of sleep deprivation was enough to promote amyloid-beta accumulation [82]. Sleep deprivation thus appears to be an important risk factor for neurodegenerative illness that should ideally be identified by both general practitioners and medical specialists. Sleep deprivation alters the expression of AQP4 [83]. Certain AQP4 single nucleotide polymorphisms (SNPs) in people were associated with poor self-reported sleep quality and an elevated amyloid load [83]. This shows that genetic variations in AQP4 can affect not just amyloid buildup but also sleep quality. Chronic insomnia patients reported greater CSF levels of amyloid-42 than regularly sleeping people, similar to the effects seen after acute sleep deprivation [84].

### 3.5. Imaging and Intervention

As we briefly discussed, investigating neurofluids and the glymphatic system is very challenging from a technical perspective, even more so if we consider the goal to be a non-invasive in vivo method of assessing glymphatic function in health and disease.

Overall, the study of neurofluids has not been lacking in its arsenal. Several methods in magnetic resonance (MR) imaging exist that can help visualize contrast agent distribution and extravasation, including BBB disruption sites and subpial-to-subarachnoid leakage. Phase contrast MR studies of CSF flow are already a staple both in research and in clinical practice, helping in assessing and quantifying the pulsatile CSF flow and its hyperdynamic states in a non-invasive manner. Perfusion MRI, both contrast and non-contrast, provides valuable information on the hemodynamic state of the parenchyma, despite the relatively low spatial resolution. Neurosonology can assess vasoreactivity but also tissue compliance [85].

Despite that, only indirect methods exist to study some components of the glymphatic itself [86]. Tractography of the perivascular space (diffusion tensor imaging along the perivascular spaces, DTI-ALPS) may provide an indirect measure of glymphatic integrity and activity (Chamonix) and magnetic resonance elastography provides an examiner-independent assessment of brain compliance (and indirectly, the ability of solutes to drain through the extracellular space). The viscoelasticity of the brain indeed ties particularly well with the effect of sleep-dependent glymphatic cycling [79,87]. The diagnostic neuroimaging of glymphatic function with such “glymphograms” may give a method for predicting the likelihood of developing proteinopathies as well as a method for evaluating the efficacy of glymphatic-flow-directed treatments when they are developed.

Until then, getting a good night’s sleep is the most reliable way to maintain effective glymphatic clearance [52,77]. Waste clearance systems that work properly protect against the accumulation of hazardous waste products like amyloid-beta and amyloid precursor protein, so the proper operation of this may even postpone or prevent the beginning of neurodegenerative illnesses like AD [67]. Future health initiatives may make the upkeep of these clearance systems a priority. Many waste clearance studies underline the need for sleep. Sleep deprivation is linked to inflammation, BBB disruption, and glymphatic dysfunction, all of which have direct consequences on neurodegeneration [80]. Maintaining a good sleep pattern and engaging in daily physical exercise to boost cardiovascular health are both very straightforward ways to maintain healthy vasculature and improve waste clearance [83]. Voluntary exercise boosted waste clearance, lowered A levels, and improved synaptic integrity in elderly rodents. Because there was no link with BBB permeability, it suggests that the enhanced clearance was caused by the glymphatic system. In addition, mice in the exercise group showed better spatial memory, suggesting that voluntary exercise can improve cognitive function by enhancing glymphatic clearance [80,83]. Recent breakthroughs in neuroimaging have enabled a variety of ways for mapping the human glymphatic system and assessing its functional competency in the setting of disease, as well as the implications of these technologies on sleep-dependent glymphatic cycling [79]. The diagnostic neuroimaging of glymphatic function by such “glymphograms” may give a method for predicting the likelihood of developing proteinopathies as well as a method for evaluating the efficacy of glymphatic-flow-directed treatments when they are developed. Until then, getting a good night’s sleep is the most reliable way to maintain effective glymphatic clearance [77,88]. Waste clearance systems that work properly protect against the accumulation of hazardous waste products like A and may even postpone or prevent the beginning of neurodegenerative illnesses like Alzheimer’s [67]. Future health initiatives may make the upkeep of these clearance systems a priority. Many waste clearance studies underline the need for sleep. Sleep deprivation is linked to inflammation, BBB disruption, and glymphatic dysfunction, all of which have direct consequences on neurodegeneration [80,89]. Maintaining a good sleep pattern and engaging in daily physical exercise to boost cardiovascular health are both very straightforward ways to maintain healthy vasculature and improve waste clearance [83,89]. Voluntary exercise boosted waste clearance, lowered A levels, and improved synaptic integrity in elderly rodents. Because there was no link with BBB permeability, it suggests that the enhanced clearance was caused by the glymphatic system. In addition, mice in the exercise group showed better spatial memory, suggesting that voluntary exercise can improve cognitive function by enhancing glymphatic clearance [80,83].

## 4. Discussion

The involvement of sleep in the physiology of the various types of memory and the deleterious effect that the disruption of sleep has on memory is well established. Senescence, on the other hand, affects both phenomena through several aspects of aging and neurodegeneration. In this review, we aim to present as comprehensive of an overview of this knowledge as possible, with the addition of the glymphatic system as a superimposed network of vital physiological and pathological interactions.

The glymphatic system is still an incompletely understood cerebral mechanism that is essential in neurofluid circulation, tissue homeostasis, neuroimmunology regulation, and several other functions of the central nervous system [77,88]. A number of pathological processes, however, can disrupt the normal functioning of the glymphatic system, resulting in detrimental effects on brain health [8,9]. Aging and other factors associated with a vascular injury can disrupt normal arterial pulsatility, which plays a key role in neurofluid circulation [43,49,90]. The loss of the AQP4 concentration around the periarterial space can also occur as a result of aging, cerebrovascular accidents, and neuro-autoimmune processes, which further compromise glymphatic function [10,75]. Similarly, sleep disorders can inhibit the tissue compliance changes that permit ISF flow and waste advection that seems to take place during sleep, thereby impairing the glymphatic system’s capacity to remove waste and solutes [13]. Thus, the effect of aging on sleep is yet another way that aging affects the function of the glymphatic system.

**Figure 1:**
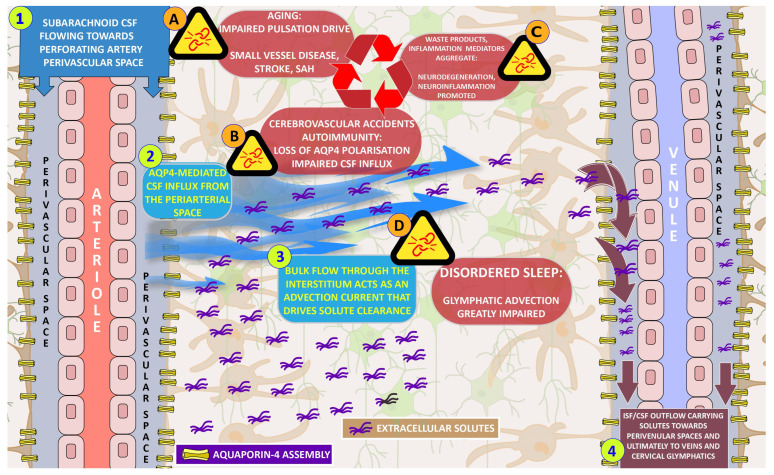
A simplified overview of the glymphatic system. Numbered steps (1–4) represent the main physiological processes. Alphabetically numbered text boxes represent pathological processes that disrupt the glymphatic mechanism. 1. The paravascular space conduction of cerebrospinal fluid along penetrating arterioles. 2. AQP4 channels situated at the astrocytic end feet lining the perivascular space facilitate the transport of fluid towards the extracellular space. 3. A net flow of ISF carries extracellular solutes in an advective current towards the postulated draining sites. 4. The draining of ISF to the perivenular fluid and from there to dural and cervical lymphatics and/or venous reabsorption sites. A. Aging and other concomitant factors of vascular injury disrupt normal arterial pulsatility. B. The loss of the AQP4 concentration around the periarterial space as a result of cerebrovascular accidents and neuro-autoimmune processes. C. Sleep disorders inhibit ISF flow augmentation that occurs in normal sleep. D. Accumulation of harmful waste and pro-inflammatory mediator molecules as a result of glymphatic dysfunction. The interconnectedness of pathological processes, noted with red arrows, results in positive feedback loops of damage amplification. This figure expands on a diagrammatic illustration by Illiff et al., 2012 [52].

The degree to which neurofluid circulation is compartmentalized or not is yet to be determined. However, our current understanding is that the disruption of any point in the glymphatic process can have a profound negative effect in solute clearance, at least in an experimental setting [10]. In this way, any injury can have a dynamic impact, creating positive feedback loops that gradually enhance dysfunction, as we attempt to illustrate in our diagram (Figure 1). In the end, glymphatic dysfunction can lead to the accumulation of toxic waste and pro-inflammatory molecules, which contribute to neuroinflammation and neurodegeneration, resulting in memory dysfunction, although this loss may be limited to one form of memory, and the various dementia syndromes with their concomitant amnestic manifestations and overall cognitive decline [10,18,20,30].

Understanding the connection between memory function and the integrity of the glymphatic system, as well as the effects of sleep, aging, and neuroinflammation, can aid in the development of therapeutic strategies for neurodegenerative disorders [23,44]. A further investigation of the processes governed by the glymphatic system could cast light on the relationship between aging and memory impairment. However, it is important to observe that some questions have been raised about the existence and significance of the glymphatic system’s function. Uncertainty still surrounds the degree to which the glymphatic system directly influences memory formation and retrieval. Studies have demonstrated that the glymphatic system plays a role in clearing metabolic debris and solutes associated with neurodegenerative diseases [56]; however, its direct effect on memory processes such as consolidation and recall is not well understood. Some researchers hypothesize that the glymphatic system indirectly influences memory by maintaining brain homeostasis and reducing neuroinflammation, both of which can impact cognitive function [85]. Nonetheless, the precise mechanisms and extent of direct involvement are still under investigation. They contend that the observed migration of CSF and solutes along perivascular spaces may be the result of experimental artefacts rather than a genuine physiological system [57]. However, numerous studies have provided evidence supporting the existence of the glymphatic system and its essential functions, such as waste clearance and solute transport [52]. The precise spectrum of solutes removed by the glymphatic system continues to be a source of controversy. Although studies have demonstrated the clearance of molecules such as amyloid-beta and tau proteins [58], it is not yet known whether the glymphatic system can effectively clear other detritus and toxic molecules. To clarify the selectivity and capacity of the glymphatic system for various solutes, additional research is required. In addition, various research groups may employ diverse techniques and methodologies to study the glymphatic system [79,85], resulting in contradictory findings. This can make drawing definitive conclusions and reconciling contradictory findings difficult.

## Figures and Tables

**Table 1:** Memory types.

Long-Term Memory (LTM)	Short-Term Memory (STM)	Working Memory (WM)
Storage and Retrieval of information over an extended period of time	Temporary storage and manipulation of informationLimited Capacity	Responsible for temporarily holding and actively manipulating information
Unlimited Capacity Two main types: Explicit/Declarative Memory (Facts, concepts, general knowledge) Implicit/Non-declarative Memory (motor skills, practices) Main location: Hippocampus, amygdala	A small amount of information for a brief duration of data (20–30 s) Processing of incoming sensory information Main location: The lower part of the temporal lobe	Limited Capacity Interact with attentional control, information updating, and mental manipulation of stored information Main location: Frontoparietal brain regions and prefrontal, cingulate, and parietal cortices

## Data Availability

Not applicable.
